# Disease‐inclusive exercise classes improve physical fitness and reduce depressive symptoms in individuals with and without Parkinson's disease—A feasibility study

**DOI:** 10.1002/brb3.2352

**Published:** 2021-09-02

**Authors:** Tim Stuckenschneider, Vera Abeln, Tina Foitschik, Thomas Abel, Maria Cristina Polidori, Heiko K. Strüder

**Affiliations:** ^1^ Institute of Movement and Neurosciences German Sport University Cologne Germany; ^2^ Geriatric Medicine, Department for Health Services Research School of Medicine and Health Sciences Carl von Ossietzky University Oldenburg Germany; ^3^ Aging Clinical Research, Department II of Internal Medicine and Center for Molecular Medicine Cologne, Faculty of Medicine and University Hospital Cologne University of Cologne Cologne Germany; ^4^ Cologne Excellence Cluster on Cellular Stress‐Responses in Aging‐Associated Diseases (CECAD) Faculty of Medicine and University Hospital Cologne University of Cologne Cologne Germany

**Keywords:** cognition, depression, disease‐inclusive exercise, insulin‐like growth factor I, Parkinson's disease, physical fitness

## Abstract

**Background and purpose:**

Exercise is an adjunctive treatment in the management of Parkinson's disease (PD), but barriers such as health status, fear of overexertion, and lack of transportation to the location prevent regular exercise participation. Disease‐inclusive exercise classes may offer an opportunity to make exercise more accessible for older adults with and without diseases. However, the efficacy of such heterogenous exercise classes is still widely unknown. Therefore, it was the aim of this study to analyze the feasibility of disease‐inclusive exercise classes in older adults with and without PD.

**Methods:**

Twenty‐one older adults (healthy older adults (HOA): *n* = 13; PD: *n* = 8) completed an 8‐week multimodal exercise intervention in supervised group sessions. Exercise classes lasted 60 min with the goal of two participations a week. We assessed physical fitness (timed up and go test [TUG], 6‐minute walking test [6MWT], single leg stance), depressive symptoms and cognitive functions, and we determined growth factors (BDNF & IGF‐1) before and after the intervention to determine the effects and by that, the feasibility of a disease‐inclusive exercise program. Repeated measures ANOVA were used to establish changes.

**Results:**

TUG and 6MWT improved significantly after the training in both HOA (*p* = .008; *p* < .001) and individuals with PD (*p* = .024; *p* < .001). Furthermore, individuals with PD increased single leg stance left (*p* = .003). HOA (*p* = .003) and individuals with PD (*p* = .001) decreased their depressive symptoms between pre‐ and post‐test significantly. Whereas growth factors tended to improve, no differences in cognitive functions were revealed.

**Conclusion:**

Disease‐inclusive multicomponent exercise improved physical functions and reduced depressive symptoms independent of health status. This should encourage exercise providers, researchers, and clinicians to further investigate disease‐inclusive exercise, because they may have an important social impact and represent a more inclusive society.

## Graphical abstract

Older adults with and without Parkinson's disease benefit from disease‐inclusive exercise classes by reducing depressive symptoms and increasing physical fitness. Further, these classes may set an example for an open and inclusive society.

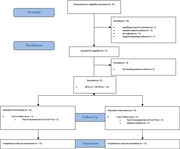

## 1 INTRODUCTION

Cognitive and physical functions follow a similar pattern throughout a person's lifespan: they are developed during childhood, maintained during maturity, and slowly decline during old age (Craik & Bialystok, 2006). Therefore, a generally aged population, in which one‐third will be 65 years or older by 2100 (Lutz et al., 2008), presents a challenge for healthcare systems worldwide. Besides the “normal” age‐related decline, age is a dominant risk factor for a number of diseases that affect cognitive and physical functions. The two most common neurodegenerative diseases are Alzheimer's disease (AD), whose key symptom is a progressive cognitive decline, and Parkinson's disease (PD), which causes motor (e.g., bradykinesia, tremor, rigor, postural instability) and non‐motor symptoms (e.g., cognitive dysfunction, depression, anxiety; Aarsland et al., 2017).

A physically active lifestyle and exercise are important factors to maintain physical and cognitive functions during old age, as well as to prevent aging‐associated diseases (Northey et al., 2018; Pahor et al., 2014). Regular physical exercise promotes angiogenesis, neurogenesis and synaptic plasticity (Churchill et al., 2002; Isaacs et al., 1992; Vaynman & Gomez‐Pinilla, 2006) and has the potential to protect cognitive function with ageing (Gomes‐Osman et al., 2018; Norton et al., 2014; Tolppanen et al., 2015). Furthermore, exercise is recommended as a promising treatment approach to slow down and/or alleviate symptoms of diseases like AD, its prodromal stage mild cognitive impairment, and PD (Erickson et al., 2019; Fox et al., 2018; Petersen et al., 2018; Salgado et al., 2013; Schenkman et al., 2018). As such, exercise needs to be accessible to older adults whether they live with or without disease.

Previous studies reported that older adults often quote health status as the major barrier for exercise participation, with lack of adequate information about exercise classes, fear of overexertion, and lack of transportation to the location cited as additional reasons (Bethancourt et al., 2014; Moschny et al., 2011). These and more barriers have also been named in another article, in which the authors concluded that raising awareness of the beneficial effects of exercise is most important to engage individuals with PD in exercise and that experts specifically trained for patients suffering from PD are needed for these classes (Schootemeijer et al., 2020). Even though it may be argued that specific disease characteristics such as gait disturbances or a poor balance could be better addressed in target exercise classes, it may limit accessibility, especially in rural settings where fewer PD specific exercise classes may be available.

In a recent article, Bechard et al. (2020) discuss the possibility of dementia‐inclusive exercise classes and conclude that willingness for such classes exists among exercise providers. Disease‐inclusive exercise classes may offer an opportunity to make exercise more accessible for older adults with and without diseases, and may further act as an example for an open and inclusive society. However, the efficacy of such heterogenous exercise classes is still widely unknown, as previous research has often targeted only specific populations such as healthy older adults, individuals with cognitive impairment or with PD. Especially, Parkinson's disease‐inclusive exercise classes appear challenging due to rather severe motor symptoms experienced by some individuals throughout the course of PD. Therefore, the aim of this pilot study was to analyze the feasibility of an 8‐week multimodal exercise intervention in individuals with and without PD. We hypothesized that individuals with and without PD would reach target exercise frequency and that the occurrence of adverse events would be similar between these two groups. Further, we hypothesized that all individuals, independent of health status, would improve physical and cognitive functions.

## 2 METHODS

### 2.1 Trial overview

This non‐randomized pilot feasibility study included an 8‐week multimodal supervised exercise intervention, which was conducted at the German Sport University, Cologne, Germany. Participants were recruited through leaflets, local support groups, advertisement on the homepage as well as through a research newsletter of the German Sport University. The ethics committee of the German Sport University, Cologne, Germany, approved the study (reference number: 172/2019). All participants provided written informed consent to participate in accordance with the provisions of the Declaration of Helsinki. Participants were recruited between February 2020 and August 2020.

### 2.2 Participants and study procedure

Adults aged 60 years or older, either diagnosed with PD or without any major disorder (healthy older adults = HOA), were recruited. Participants were initially interviewed via telephone and were required to meet the following eligibility criteria: (1) an age of 60 years or above, (2) the absence of any other major comorbidity such as dementia or depression, (3) sufficient physical ability and medical clearance to allow performance of exercise training, (4) a Montreal Cognitive Assessment (MoCA) score above 18, (5) adequate visual and auditory acuity to complete neuropsychological testing, (6) capacity to provide written and dated informed consent. Additionally, for participants with PD, the diagnosis of idiopathic PD and its severity were established by the clinical neurologist treating the patient under routine care. The neurologist provided written consent of the diagnosis of idiopathic PD and its severity to the study team. Participants with PD were able to participate in the project with a disease severity between 1 and 4 on the modified Hoehn and Yahr disease rating scale (Goetz et al., 2004).

Exclusion criteria were: (1) a diagnosis of AD or any other type of dementia, (2) engagement in moderate‐intensity exercise training for more than 30 min, three times per week, during the past 2 years, (3) enrollment in any investigational drug study, any major psychiatric disorder (a clinical diagnosis of major depressive disorder, bipolar, or schizophrenia), (4) past history of brain damage, including significant trauma, stroke, hydrocephalus, mental retardation, (5) carotid stent or severe stenosis, (6) history of myocardial infarction within previous year, (7) congestive heart failure (New York Heart Association Class II, III or IV), (8) unstable cardiac, renal, lung, liver, or other severe chronic disease, (9) type 2 diabetes mellitus with hypoglycemia in the last 3 months, (10) significant history of alcoholism or drug abuse within last 10 years.

Individuals with PD participated in this study while on medication. Twenty‐one participants completed the study. Participant characteristics at baseline including age, sex, education, height, weight, number of medications used, and disease severity are presented in Table 1.

**TABLE 1:** Group demographics (means & 95% confidence interval)

	PD	HOA	*p*‐value
*N*	8	13	
Females/males (*n*)	5/3	8/5	(χ^2^) .584
Age (years)	71.38 [66.89–75.86]	69.00 [65.32–72.68]	.301
Education (years)	15.8 [12.9–18.6]	18.0 [16.3–19.7]	.113
Height (cm)	169.9 [164.5–175.3]	171.4 [166.3–176.5]	.670
Weight (kg)	76.88 [60.7–93.0]	81.62 [68.8–94.4]	.613
MoCA	25.6 [23.8–27.5]	26.3 [25.1–27.5]	.320
No. of medications used	4.88 [2.07–7.68]	1.46 [0.46–2.47]	**.006**
Disease severity (mod. Hoehn and Yahr)	2.2 [1.4–2.9]	/	
Stage 1.5, *n*	3	/	
Stage 2, *n*	3	/	
Stage 3, *n*	1	/	
Stage 4, *n*	1	/	
Exercise sessions^a^	2.3 [1.7–2.8]	3.0 [2.5–3.4]	**.045**
CES‐D	17.3 [12.9–21.6]	15.0 [11.7–18.3]	.362

Abbreviations: PD, Parkinson's disease; HOA, healthy older adults; MoCA, Montreal Cognitive Assessment; mod, modified; CES‐D, Center for Epidemiologic Studies Depression Scale.

^a^ Average number of exercise sessions per week.

### 2.3 Intervention

Participants attended supervised instructor‐led exercise classes conducted at an indoor gym at the German Sport University. Eight exercise sessions lasting 60 min each were offered from Monday to Friday over the course of 8 weeks. The multicomponent exercise training consisted of 20 min of coordination exercises (e.g., balance or dual‐task exercises), followed by 20 min of resistance training (using one's own body weight and small equipment such as medicine balls) and 20 min of aerobic exercise training (e.g., walking or running exercises). Coordination exercises included, for example, standing on even or uneven ground with eyes open or closed (balance), sitting on a chair while lifting one's toes or heels and throwing and catching a ball at the same time (dual task). Resistance exercises were, for example, wall pushups, holding medicine balls with outstretched arms, squats, or crunches. Exercise intensity was monitored using Borg's Rating of Perceived Exertion Scale (RPE), which is a scale from 6 (“no exertion”) to 20 (“maximal exertion”) (Borg, 1998). Participants had a target RPE of at least 13 while performing resistance and aerobic exercises. Participants were asked to participate in at least two exercise classes per week and class attendance and adherence were monitored by class instructors. If needed, participants were allowed to use a walking stick during the exercise classes or perform exercises while holding on to wall bars. Exercise classes were led and designed by sports scientists experienced in working with individuals with PD before. During the exercise classes, chairs were distributed throughout the indoor gym to allow participants to sit down if they were feeling tired or shaky.

### 2.4 Primary outcome measures

Primary outcome measures included adherence (average number of participations a week) and adverse events, which were recorded in study‐specific adverse event forms. Further, participants were asked if they generally liked to continue with the disease‐inclusive exercise training after completion of the follow‐up measurements.

### 2.5 Secondary exploratory outcome measures

All secondary outcomes were measured at baseline and after 8 weeks of exercise training. Outcome assessors were not blinded. The participants underwent a brief neuropsychological assessment battery, comprising six different tests using German‐language versions: the Montreal Cognitive Assessment (Nasreddine et al., 2005), the Trail Making Test (TMT) parts A and B (Tombaugh, 2004), letter and semantic verbal fluency (Lezak et al., 2012), digit span forward and backward (Wechsler, 2008) as well as the verbal learning and memory test (VLMT) (Helmstaedter et al., 2001).

The MoCA is a screening tool for global cognitive function. Besides its total score, MoCA memory index score was calculated and used as another outcome measure (Julayanont et al., 2014; Nasreddine et al., 2005). TMT A and B assess speed of processing and executive function, and the time taken to complete each test was measured as the outcome (Tombaugh, 2004). To determine letter and semantic verbal fluency, participants were given 1 min to produce as many unique words as possible either starting with a given letter or unique words within a category. A participant's score was the number of unique correct words in each task (Lezak et al., 2012). Digit span was used to quantify working memory. We used digit span forwards and backwards and calculated the correct responses given in each of the tasks (Lezak et al., 2012). VLMT served as a measure of verbal memory. Outcomes of the VLMT included total learning over five trials and delayed free recall as well as recognition (measured 30 min after the learning phase) (Helmstaedter et al., 2001).

The Center for Epidemiologic Studies Depression Scale (CES‐D) was used to measure depressive symptoms. The CES‐D consists of 20 questions and is a self‐report measure about symptoms experienced over the previous week. Items are scored from 0 to 3, with 3 indicating the highest frequency of symptoms experienced in the past week. Total scores range from 0 to 60, with lower scores indicating less symptoms (Radloff, 1977). A score of ≥16 has frequently been named as a cut‐off for the detection of depressive symptoms; however, a score of ≥20 may provide a better trade‐off between sensitivity and specificity (Schrag et al., 2007; Vilagut et al., 2016).

Physical fitness was assessed using a 6 min walking test (6MWT) (Falvo & Earhart, 2009), 30 s chair stand test (Jones et al., 1999), hand grip strength test measured in kilograms (Roberts et al., 2011), the timed up and go test (TUG; 3‐meter distance) (Podsiadlo & Richardson, 1991) and the balance tests from the Short Physical Performance Battery (Guralnik et al., 1995), as well as a single leg stance test (Springer et al., 2007). During the 6MWT, participants were asked to walk as far as they could for 6 min on a 100‐meter track. Participants were advised to slow down, stop, or use walking aids if needed. Total distance walked was measured in meters.

The 30 s chair stand determined lower limb strength. Participants were asked to stand up and sit down as often as possible over 30 s. Hand support was not allowed. TUG was applied to quantify functional mobility and walking speed. Mean time of three trials needed to walk back and forth was measured. Hand‐grip strength for right and left hand was measured using a Jamar Digital Dynamometer. Balance was assessed using three different positions (side‐by‐side stand, semi‐tandem stand, and tandem stand), which participants were asked to hold for 10 s. For the first two positions, the score was 0 or 1, reflecting the ability to either hold (1) or not hold (0) the position for 10 s. For the tandem stand, the score was 0‐1‐2 depending on the time the individual was able to be in this position (0 = less than 3 s; 1 = between 3 and 9.99 s; 2 = 10 s). Furthermore, participants were asked to stand on a single leg for 30 s with the time holding this position being recorded.

Ten milliliters of venous blood was collected at baseline and then after the end of the intervention before the cognitive testing session. Subjects were not fasted, but were asked to avoid any caffeine before blood collection and fill out breakfast protocols to ensure they ate a similar breakfast at pre‐ and post‐test. Further, subjects were asked to avoid any kind of exercise before blood collection. Blood was drawn between 08:30 am and 10:00 am. Blood samples were kept at room temperature for an hour to allow clotting before they were centrifuged at 3000 rpm at 4°C for 10 min. Serum was then harvested, aliquoted, and stored at −80°C until analysis. Serum levels of IGF‐1 and serum levels of BDNF were quantified per manufacturer instructions using human ELISA kits (Bio‐Techne GmbH, Quantikine^®^ Human IGF‐1 Immunoassay [Catalognr.: DG100B]/Human free BDNF immunoassay [Catalognr.:DBNT00], R&D Systems Inc., USA). A standard curve of recombinant BDNF and IGF‐1 was run on each plate. Samples and standards were run in duplicate.

### 2.6 COVID‐19 pandemic

**FIGURE 1:**
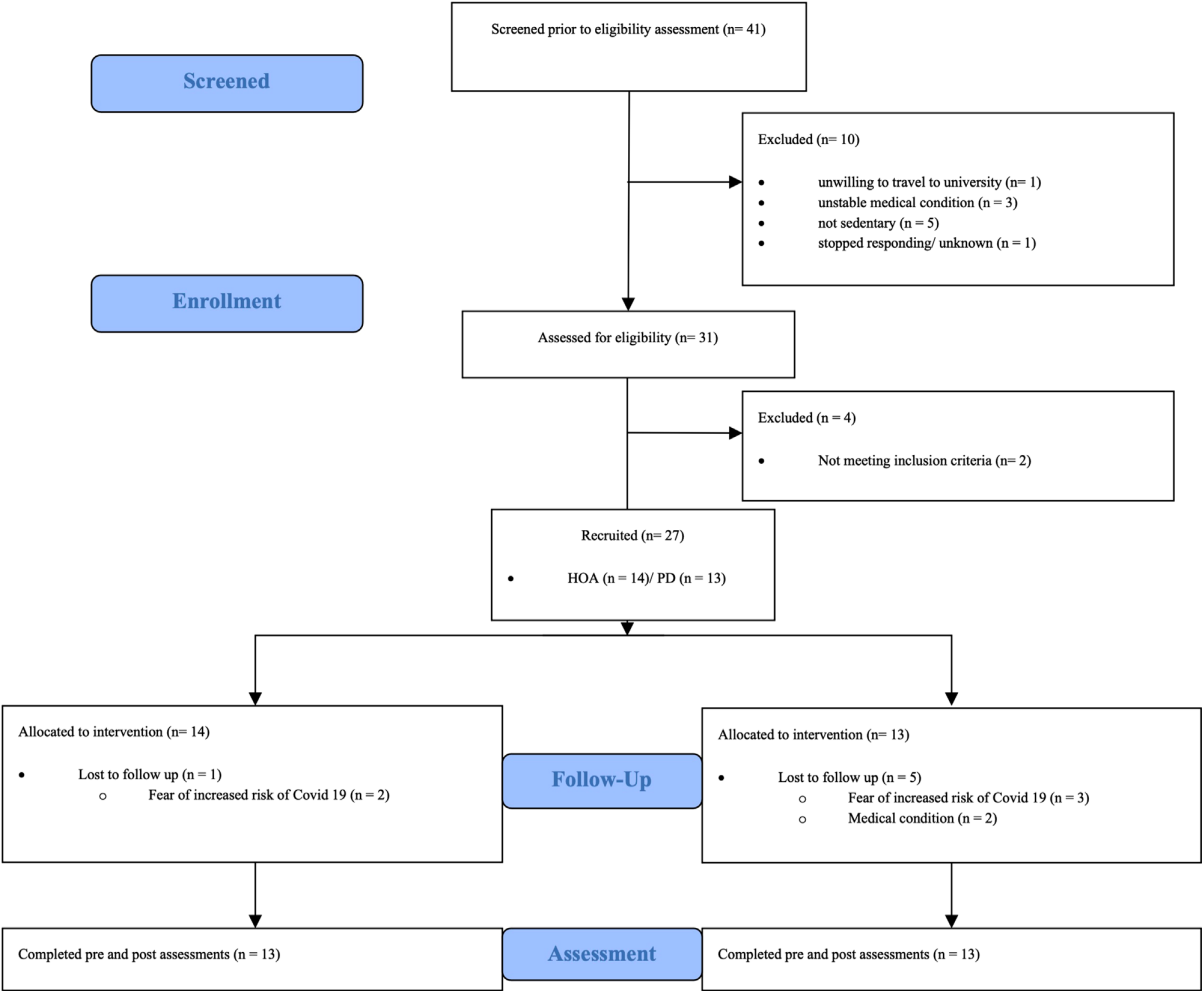
Participant flow chart

Recruitment started in February 2020 with a recruitment aim of 30 participants (PD: *n* = 15; HOA: *n* = 15). Exercise classes for individuals with PD are not allowed to exceed 15 participants to be supported by public healthcare. Therefore, a total of 30 participants, combined with exercise classes every day of the week, allowed us to follow recommendations of exercise providers and healthcare officials. Twenty‐seven participants (PD: *n* = 13; HOA: *n* = 14) had successfully been recruited at the start of March. Due to the outbreak of COVID‐19 and associated local restrictions, the study had to pause from March 14 and was restarted in August 2020. All participants started a new cycle of 8 weeks of training and repeated baseline measures regardless of their training at the start of March. Due to the then ongoing restrictions, capacity of the exercise classes had to be limited to a maximum of 12 participants. Therefore, no new participants were recruited. At the restart of the study, 3 participants with PD and 1 participant of the HOA group dropped out due to fear of higher infection in indoor exercise classes. Two more participants with PD dropped out after completing 4 weeks of training due to the diagnosis of skin cancer and suffering from a stroke. Therefore, 13 HOA and 8 individuals with PD completed the intervention and all assessments (Figure 1). Post‐tests were conducted in October 2020 before another nationwide lockdown due to the COVID‐19 pandemic.

### 2.7 Statistical analysis

IBM SPSS Statistics version 27 was used for all analyses with α set at 0.05. Two‐tailed independent samples *t*‐tests or Mann–Whitney *U*‐test for non‐normally distributed data (age, number of medications used, average number of exercise sessions per week) were employed to assess group (HOA, PD) differences in baseline characteristics. A repeated measures of variance analysis (ANOVA) was carried out with the within‐subjects factor time (pre‐ and post‐test) and the between‐subjects factor group (HOA and PD). Variables pertaining to cognitive function, physical fitness assessment, blood analyses, and quality of life were compared using the aforementioned analysis. In case of significant time, group, or interaction effects, post hoc pairwise comparisons were conducted using Bonferroni correction for multiple pairwise comparisons.

Due to the lack of an equivalent non‐parametric analysis and due to ANOVA being relatively robust to violations of the normal distribution assumption (Schmider et al., 2010), this analysis approach was used for all outcomes. Data are presented as means and 95% confidence intervals within brackets.

## 3 RESULTS

### 3.1 Baseline demographics

Age, cognitive function assessed with MoCA, education, height, and weight did not differ between HOA and PD at baseline. Furthermore, the proportion of men and women was similar in the two groups. Participants with PD had an average disease severity of 2.2 according to the HY scale and used significantly more medication (*p* = .006) than HOA (Table 1).

### 3.2 Primary outcome

Whereas both groups reached the mean target exercise frequency of two exercise sessions a week, HOA participated significantly more often in the exercise classes than participants with PD (*p* = .045). No serious adverse events associated with the study and its training program occurred. No adverse events such as falls or overexertion occurred during the training program; however, one individual with PD and two HOA persons had to pause during respective single exercise sessions due to vertigo. All participants completing the intervention responded that they would like to continue with the disease‐inclusive exercise training.

### 3.3 Cognitive function and depressive symptoms

Besides a group difference for digit span backwards (*p* = .038), repeated measures ANOVA did not reveal changes over time or between the groups for the other neuropsychological tests. Bonferroni‐corrected post hoc pairwise comparisons showed that individuals with PD performed significantly worse in digit span backwards at pre‐test (*p* = .028) but not post‐test (*p* = .094) compared to HOA. Symptoms of depression were significantly reduced between pre‐ and post‐test (F_1, 19_ = 25.297; *p* < .001) with no differences between the groups (F_1, 19_ = 0.454; *p* = .508) or an interaction of group*time (F_1, 19_ = 0.713; *p* = .409). Bonferroni‐corrected post hoc pairwise comparisons revealed statistically relevant reductions in depressive symptoms in both HOA (*p *= .003) and individuals with PD (*p* = .001) between pre‐ and post‐test. Full results (mean + 95% CI) are presented in Table 2.

**TABLE 2:** Results of repeated measures ANOVA for neuropsychological tests and depressive symptoms (means & 95% confidence interval)

	PD		HOA		*p*‐value		
	Pre	Post	Pre	Post	Time	Group	Time*Group
MoCA (score)	25.38 [25.09–27.53]	25.00 [23.05–26.95]	26.31 [25.09–27.53]	26.08 [24.72–27.44]	.354	.287	.823
MoCA‐MIS (score)	9.88 [7.33–12.42]	9.63 [6.28–12.97]	10.92 [9.19–12.66]	10.92 [9.12–12.73]	.857	.360	.857
TMT A (time in s)	42.2 [31.4–53.0]	43.6 [20.6–66.7]	39.72 [32.3–47.1]	38.37 [32.2–44.5]	.991	.527	.714
TMT B (time in s)	128.6 [94.4–162.7]	131.3 [64.2–198.4]	105.4 [54.1–156.7]	100.4 [67.6–133.2]	.925	.355	.742
Digit span – FW (correct responses)	8.00 [6.52–9.48]	7.88 [6.58–9.17]	8.54 [7.34–9.74]	8.92 [7.98–9.86]	.613	.297	.325
Digit span – BW (correct responses)	4.88 [3.74–6.01]	5.38 [4.04–6.71]	6.38 [5.51–7.26]	6.69 [5.66–7.72]	.174	**.038**	.740
VLMT – immediate (number of words)	37.00 [28.51–45.49]	38.50 [31.78–45.22]	39.46 [32.44–46.49]	41.77 [34.74–48.80]	.398	.515	.856
VLMT – delayed (number of words)	5.88 [3.21–8.54]	5.00 [2.51–7.49]	7.54 [5.14–9.94]	7.46 [5.53–9.40]	.398	.167	.477
VLMT – recognition (number of words)	11.75 [9.44–14.06]	11.88 [8.61–15.14]	13.08 [11.51–14.65]	13.08 [11.72–14.44]	.920	.269	.920
Verbal fluency – letter (number of words)	12.88 [9.62–16.13]	13.00 [10.52–15.48]	14.31 [11.30–17.32]	14.74 [11.50–17.98]	.648	.433	.800
Verbal fluency – category (number of words)	22.63 [16.09–29.16]	20.88 [16.67–25.08]	21.69 [17.77–25.62]	23.08 [17.81–28.34]	.887	.836	.234
CES‐D (score)	17.3 [12.9–21.6]	10.1 [6.2–14.1]	15.0 [11.7–18.3]	9.9 [7.5–12.4]	**<.001**	.508	.409

Abbreviations: PD, Parkinson's disease; HOA, healthy older adults; MoCA, Montreal Cognitive Assessment; MIS, Memory Index Score; TMT, Trail Making Test; FW, forward; BW, backward; VLMT, Verbal Learning and Memory Test; CES‐D, Center for Epidemiologic Studies Depression Scale; s, seconds.

### 3.4 Physical fitness

Significant time effects were found for 6MWT distance (F_1, 19_ = 14.025; *p* = .001), TUG test (F_1, 19_ = 102.851; *p* < .001), as well as 30 s chair stand (F_1, 19_ = 290.466; *p* < .001). Further, ANOVA showed a significant group effect in 6MWT distance (F_1, 19_ = 6.588; *p* = .019). Post hoc pairwise comparisons revealed that both individuals with PD (*p* = .024) and HOA (*p* = .008) improved their walking distance between pre‐ and post‐test significantly. Compared to HOA, individuals with PD had a significantly lower walking distance at pre‐test (*p* = .013), but not at post‐test (*p* = .078). Both HOA (*p* < .001) and individuals with PD (*p* < .001) improved their walking speed (TUG) between pre‐ and post‐test with no differences between the groups. In line with walking speed, number of repetitions during the 30 s chair stand test were increased according to Bonferroni‐corrected post hoc tests (HOA: *p* = .008; PD = 0.001). Additionally, ANOVA revealed significant group effects in single leg stance left (F_1, 19_ = 10.683; *p* = .004) and right (F_1, 19_ = 8.637; *p* = .008) as well as a significant interaction effect group*time for single leg stance left (F_1, 19_ = 10.683; *p* = .004). Individuals with PD had a significantly worse balance than HOA on the left (*p* = .002) and right leg (*p* < .001) at pre‐test, but no differences between the groups were found at post‐test. Further, individuals with PD significantly improved their balance for single leg stance left (*p* = .003) between pre‐ and post‐test. Hand grip strength did not change in or between the groups (Table 3).

**TABLE 3:** Results of repeated measures ANOVA for physical fitness measures (means & 95% confidence interval)

	PD		HOA		*p*‐value		
	Pre	Post	Pre	Post	Time	Group	Time*Group
Single leg stance – right (time in s)	9.0 [−1.6–19.6]	14.4 [4.4–24.5]	25.7 [20.2–31.3]	23.2 [17.4–29.1]	.461	**.008**	.057
Single leg stance – left (time in s)	2.4 [0.2–4.6]	13.9 [4.8–23.1]	23.5 [17.5–29.5]	21.1 [14.4–27.8]	.045	**.004**	**.001**
Distance 6MWT (in meter)	480.6 [375.2–586.0]	570.0 [493.8–646.2]	572.5 [536.7–608.2]	655.2 [578.2–732.1]	**.001**	**.019**	.917
Hand grip strength – right (kg)	32.0 [24.4–39.6]	32.1 [23.6–40.7]	34.1 [27.8–40.4]	33.9 [ 27.8–40.1]	.979	.505	.803
Hand grip strength – left (kg)	30.7 [22.6–38.8]	31.3 [23.6–39.0]	30.1 [24.1–36.1]	31.7 [26.0–37.4]	.169	.760	.752
30 s chair stand (number of repetitions)	10.5 [8.1–12.9]	13.8 [11.8–15.7]	13.0 [10.9–15.1]	14.9 [12.1–17.6]	**<.001**	.254	.179
TUG (time in s)	9.6 [8.3–10.9]	6.8 [6.3–7.2]	8.7 [8.0–9.4]	6.6 [6.1–7.1]	**<.001**	.197	.268

Abbreviations: PD, Parkinson's disease; HOA, healthy older adults; 6MWT, 6‐minute walking test; s, second; TUG, Timed Up and Go.

### 3.5 Growth factors

**FIGURE 2:**
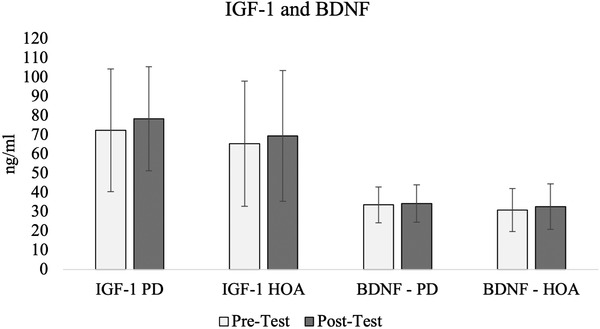
Serum levels of IGF‐1 and BDNF at pre‐ and post‐test. HOA, healthy older adults; PD, Parkinson's disease

Both groups slightly increased serum levels of IGF‐1 and BDNF after the multicomponent exercise intervention (Figure 2). Repeated measures ANOVA revealed a significant time effect for IGF‐1(F_1, 19_ = 4.826; *p* = .044), but no group (F_1, 19_ = 7.384; *p* = .797) or interaction effect (F_1, 19_ = 0.069; *p* = .797). Post hoc pairwise comparisons revealed no further differences. No time (F_1, 19_ = 0.788; *p* = .386), group (F_1, 19_ = 0.504; *p* = .486) or interaction effect (F_1, 19_ < 0.000; *p* = .985) was detected for BDNF.

## 4 DISCUSSION

This pilot study analyzed the feasibility of a disease‐inclusive 8‐week multimodal exercise intervention. Both groups reached the target exercise frequency with no serious adverse events reported. Furthermore, we identified an intervention effect on physical fitness and depressive symptoms in healthy older adults and individuals with Parkinson's disease. Growth factors tended to improve, whereas no effect was identified for cognitive function.

Both individuals with PD and HOA reached the target exercise frequency of two exercise sessions a week. Even though this exercise frequency is below current exercise recommendations for older adults (Bull et al., 2020), it appeared more manageable for older adults with little to no previous experience in exercise training and was in line with the average exercise frequency reported in previous studies (Stuckenschneider et al., 2019). In comparison, HOA exercised significantly more often than individuals with PD. This was possible as training sessions were offered on all days of the week and participants were not limited to two sessions a week. We speculate that individuals with PD either needed more time to recover between exercise sessions or had more additional appointments such as physiotherapy or speech therapy, which kept them from participating more often. As the best intensity and frequency of exercise training is still unknown, future research needs to compare different exercise prescriptions to define the most effective ways (Ellis & Rochester, 2018). No differences in adverse events between individuals with and without PD, and all participants responding at follow‐up that they would have liked to continue the disease‐inclusive exercise training, support that disease‐inclusive exercise training was not only feasible, but enjoyable for all individuals.

Physical fitness quantified by 6MWT, 30 s chair stand (lower limb strength), and walking speed quantified by TUG increased significantly after the 8‐week training period. Whereas this result is in line with existing literature, as standardized exercise training increases walking speed and distance (Rafferty et al., 2017; Stuckenschneider et al., 2015), it is encouraging that this result was achieved in the study's heterogenous sample. Therefore, the primary aim of exercise classes, which is to maintain and improve physical fitness, was met by the disease‐inclusive exercise training in our study. Besides the aforementioned physical improvements, participants with PD had a better balance after the multimodal exercise training. This emphasizes the efficacy of combined modes of exercise training, that is, multicomponent exercise training, as endurance (6MWT), strength (30 second chair stand) and coordination (balance) all improved (Stuckenschneider et al., 2019). Initial differences between the groups are due to PD affecting balance, which led to a significantly lower baseline score and thus, more room for improvement in comparison to HOA, who performed in line with reference scores at pre‐ and post‐test (Springer et al., 2007). Our results should strongly encourage exercise providers, clinicians, and researchers to design disease‐inclusive exercise classes and interventions as 8 weeks of regular exercise led to statistically relevant improvements in physical fitness independent of health status.

Cognitive function did not change after 8 weeks of training. Even though exercise training with HOA and with individuals with PD is associated with an improved cognitive function (Gomes‐Osman et al., 2018; Northey et al., 2018; Stuckenschneider et al., 2019), 8 weeks of training may not be enough to result in meaningful changes. Previous studies found relevant changes in cognition in individuals with PD after 12 weeks of training (Silva‐Batista et al., 2016; Silveira et al., 2018) and Gomes‐Osman et al. (2018) reported that 52 h of exercise are associated with improved cognitive performance in healthy older adults and those with cognitive impairment. Therefore, an extension of the intervention period is warranted in future studies as our trial showed its feasibility. Even though we did not identify any changes in cognitive function, BDNF and IGF‐1 tended to increase after 8 weeks of training. As exercise induces the expression of growth factors throughout the nervous system and as an increased bioavailability of growth factors such as BDNF and IGF‐1 might stimulate neurogenesis and angiogenesis (Knaepen et al., 2010; Maass et al., 2016; Vaynman et al., 2004), the preliminary findings of our study are in line with previous research. The increased bioavailability of growth factors may present a physiological explanation for why exercise has a neuroprotective effect and is widely recognized as an adjunct therapy in PD (Palasz et al., 2020). Szymura et al. (2020) have previously demonstrated that similar balance training induces neuroprotective mechanisms in both HOA and individuals with PD. The lack of differences between HOA and individuals with PD in our study extends these findings and suggests that disease‐inclusive exercise training may be equally effective for both groups and thus may maintain physical and cognitive functions in older adults with or without PD. The limited number of participants included in this pilot‐trial, as well as the short observation period warrant bigger studies with longer intervention periods to fully investigate the role of exercise and a potentially following increase of growth factors on cognitive function and overall brain health.

Physical inactivity increases the risk of depression in both HOA and individuals with PD (Gustafsson et al., 2015; Lee et al., 2014), who, in general, have a higher risk of suffering from depression than HOA (Wu et al., 2017). Therefore, the reduction of depressive symptoms in both inactive HOA and individuals with PD is an important outcome of our study. Schootemeijer et al. (2020) list the discomfort of seeing advancing symptoms of peers as a barrier to participate in exercise, which may not only apply for the heterogenous group of individuals with PD (range of 1–4 on the modified HY scale), but also for HOA. The reduction in depressive symptoms may indicate that a disease‐inclusive exercise training helps to overcome this barrier. However, more specific questionnaires (e.g., acceptance + meeting individual's needs) and qualitative evaluations are needed to verify this hypothesis. Additionally, overexertion and also the lack of an adequate exercise intensity have frequently been named as barriers to exercise (Bethancourt et al., 2014) and may present another major challenge of disease‐inclusive exercise classes that could increase dissatisfaction of participants and affect long‐term adherence. However, the low number of dropouts, the reduced number of depressive symptoms, and the improved physical functions of both HOA and individuals with PD in the present study do not support this notion. Again, further evaluation is needed to analyze facilitators and barriers of disease‐inclusive exercise classes.

Disease‐related stigma affects quality of life, increases the risk of depression, social isolation, and is common in PD (Hermanns, 2013; Ma et al., 2016; Salazar et al., 2019). Thus, felt (e.g., shame, embarrassment) and enacted stigma (e.g., staring, avoiding) present an important, yet often underrepresented symptom in individuals with PD. Integrating individuals with PD into formal exercise classes could have a significant social impact and reduce the stigma surrounding PD. While this improves exercise inclusivity, it could further act as an example of an open and inclusive society. Based on the reduction of depressive symptoms, it can only be speculated that disease‐inclusive exercise classes reduced stigma in participants within this trial and specific measurements on quality of life, and self‐experienced stigma are needed to provide further insight into the efficacy of disease‐inclusive exercise.

### 4.1 Limitations

The small sample‐size presents a limitation of this study and may have made it difficult to obtain significant changes for more outcomes (e.g., growth factors). Nevertheless, we identified significant changes in relevant areas which justify the need for bigger follow‐up studies and was the aim of this feasibility study. Further, the sample size did not allow the analysis of men and women separately, which limits further analysis of sex‐related differences (Rigby & Davis, 2018). However, women and men were equally distributed in HOA and individuals with PD, so that distribution within each of the groups was not different and did not further affect our results. Due to the ongoing COVID‐19 pandemic, we decided to include a smaller number of participants as more than 12 individuals were not allowed to exercise at the same time due to local restrictions. This modification enabled us to conduct our study during the pandemic, however, it may have facilitated running the exercise classes as more than 12 participants per exercise class—and with that an even more heterogeneous group—may present bigger challenges. Therefore, the ideal number of participants per class, especially in disease‐inclusive classes, warrants further evaluation in future studies. The COVID‐19 pandemic did not only affect the sample size, but also caused us to pause and delay our study. However, all participants included in the analyses engaged in 8 weeks of exercise training. As previous research has demonstrated a negative impact of the COVID‐19 pandemic on PD symptoms, depressive symptoms, and physical activity in individuals with PD (van der Heide et al., 2020), the increases in physical fitness and especially reduced depressive symptoms following the intervention emphasize the importance of exercise. As the post‐tests were conducted in October 2020 with increasing COVID‐19 numbers and another lockdown looming, it is possible that these circumstances may have influenced our results. We did not include a non‐exercising control group, which would have provided further insight into the role of exercise classes, especially during the pandemic. Additionally, indication‐specific exercise classes, that is, only HOA or only individuals with PD, should be considered in future studies to evaluate if they are as, or more, or less effective as/than disease‐inclusive exercise classes. These future studies should further aim to increase the intervention period. This is of particular importance as the progressive course of PD may complicate long‐term participation in exercise and may present more challenges for disease‐inclusive exercise classes over time. In this pilot trial, we included a rather heterogenous sample with individuals with PD ranging in disease severity from 1 to 4 on the modified HY scale. Therefore, our results support that a broad spectrum of symptoms can be addressed adequately in disease‐inclusive exercise classes.

## 5 CONCLUSION

This pilot trial shows that 8 weeks of disease‐inclusive multicomponent exercise training improved physical functions and reduced depressive symptoms in both healthy older adults and individuals with Parkinson's disease. This should be encouraging for exercise providers, researchers, and clinicians to further investigate the possibilities of disease‐inclusive exercise training. Not only the individual independent of health status benefits from such training, but disease‐inclusive exercise classes may also have an important social impact and represent an open, diverse, and inclusive society. In the context of the demographic change and increasing numbers of older adults with and without disease, an open and supportive society is of particular importance. Future trials need to address methodological limitations (i.e., sample size, intervention period, and lack of control group) of this pilot trial to verify our findings.

## CONFLICT OF INTEREST

The authors have declared that there are no conflicts of interest in relation to the subject of this study.

### PEER REVIEW

The peer review history for this article is available at https://publons.com/publon/10.1002/brb3.2352.

## Data Availability

Data can be obtained upon reasonable request from the corresponding author.
